# Transition readiness of adolescents to adult health care

**DOI:** 10.3389/fped.2023.1204019

**Published:** 2023-07-31

**Authors:** Beatrice Rodriguez Lara-Macaraeg, Avegail Cardinal, Berith Grace Bermejo

**Affiliations:** Department of Pediatrics, Baguio General Hospital and Medical Center, Baguio, Philippines

**Keywords:** adolescent transition, transition readiness assessment, Philippines, TRAQ, transition assessment

## Abstract

**Background:**

Transition in health care is a process wherein adolescents gradually prepare for and shift towards care in the adult system. An initial assessment of the readiness of these adolescents is fundamental in providing appropriate health services for them. This paper aims to determine the readiness of adolescent patients towards transitioning to adult care.

**Methods:**

This is a prospective cross-sectional study utilizing an interviewer-guided modified Transition Readiness Assessment Questionnaire (TRAQ). Sixty-three adolescents ages 15 to 18 years seen in the Pediatric Outpatient Department (OPD) of a tertiary hospital in Baguio City from July 1 to October 31, 2020 were enrolled. Frequency and percentages were used to describe the demographic data; while mean score and standard deviation determined readiness based on a Likert scale. One-way ANOVA was utilized to determine association between factors and readiness to transition.

**Results:**

For all domains of TRAQ, the mean score was 3.64, implying that they are not yet ready to transition. 49.2% belong to General Pediatrics. 44.4% belong to the Igorot ethnic groups. Most are still in High school, and majority of their parents finished High School level. Furthermore, the P-values were more than 0.05 for all variables suggesting no association between readiness to transition and the identified factors in this study.

**Discussion:**

This study showed that adolescents who had both acute and chronic illnesses, were not yet ready to transition. Provision of a platform for assisting the adolescents in their assumption of bigger roles/responsibilities for their own health care is necessary to ensure proper transitioning to adult health care.

## Introduction

Adolescence is a period of gaining of maturity that brings many changes in the physical, mental, social and emotional aspects. During these years, they have an improved ability to think abstractly and are now able to set long term goals. Each individual may progress at a different rate and have a different view of the world, including taking responsibility for their health care (1). The developmental changes that happen during adolescence have important implications for physicians who are involved in the care of these adolescents.

In Pediatrics, we attend to patients both under general pediatrics and subspecialty clinics who need guidance in terms of shifting of attending physicians, continuity of health care plans, and accepting even the subtle changes from the routine parent-guided management that they have been comfortable with for a period of time. As pediatricians in our institution, we ensure that our patients will have a continuity of doing health care plans even if after they turn 19 years old, however, there is actually no established transition care program/protocol instituted. This necessitates a proper transition in health care which is the process wherein adolescents transfer towards the adult health care system in a progressive manner (2). An effective transition from pediatric to adult health care services is an important part of high-quality care; however, it imposes a challenge to young people especially those with chronic conditions (3). Internationally, the need to provide assistance in transitioning care to adolescents and young adults had been recognized as early as 1980's in the United States of America. Comparing between developed and developing countries health policies, one difference is the lack of effective health insurance/plans for the pediatric population. Furthermore, children under 18 years old are still considered as dependents of their parents. Adding to this is the culture of “dependence” of Filipino children to their parents even after they graduate and finish school to the point that they raise their own families- they are still living under the care of their parents. Although there is already a transition clinic in the Philippine General Hospital assessing the readiness to transition among Pediatric Nephrology patients, nevertheless, there is still paucity of established transition clinics in our country (4). This points out that even though transition clinics had been well established in the western countries, developing countries like ours is still lacking programs/clinics such as this.

In the process of transition of health care, the pediatrician's role cannot be overemphasized since they are in frequent contact with the adolescents. Aside from monitoring the physical, emotional and cognitive growth of these adolescents, their neurodevelopmental level and ability to engage in their own therapeutic care is also being assessed in every clinic or hospital visit (5). In addition to the pediatric care, the adult clinicians also share the responsibility in fostering these patient's abilities to assume a more independent role in terms of their own health care management. The interaction among the pediatrician, patient and adult clinician then provides the opportunity to build a close relationship with these patients as well as with their families. With an affective transition program, the pediatricians and adult clinicians will benefit by ensuring that the patient will be ready and will receive appropriate and well-coordinated continuity of care despite changing health care providers and health protocols. Above all else, having a transition clinic will benefit the patients and their families in terms of planning of health care, both long and short term.

The goal is to establish a transition clinic; hence this study will determine the readiness to transition from pediatric to adult health care of ill adolescent patients ages 15 to 18 years old seen at the Baguio General Hospital and Medical Center (BGHMC) Pediatrics Outpatient department (OPD) by answering the Transition Readiness Assessment Questionnaire (TRAQ) within a study period of four (4) months from July 1 to October 31 2020. The specific objectives were: (1) to describe the demographic profile of adolescents enrolled in the study including age, sex, and ethnicity, educational status of patients and parents, and diagnoses; (2) to determine the readiness of the adolescent patient towards transitioning from pediatric to adult clinics with regards to ability in managing medications, appointment keeping, tracking health issues, talking with providers and managing daily activities; (3) to determine if there is an association between the factors (such as the educational status of patients, education of their parents, and their kind of illness based on subspecialty) and the patient's readiness to transition to adult health care.

## Methodology

### Research design and population of the study

The research design used was a Cross-sectional study which utilized the TRAQ as the questionnaire on readiness to transition from the pediatric to the adult care setting. The study applied total enumeration since there was paucity of patient census at the start of the COVID-19 pandemic. Included were adolescent patients ages 15–18 years old who sought consult under General pediatrics and Subspecialty clinics (Hematology-Oncology, Endocrinology, Cardiology, Nephrology, Hepato-Gastroenterology, Pulmonology, Neurology, Rheumatology and Allergology). Patients with neurodevelopmental delay or were mentally challenged were excluded in the study. Patients who refused to answer the survey form and questionnaires that were incompletely answered were also excluded.

### Study procedure

The researcher and/or research assistant (Pediatric Resident/s assigned in the OPD) obtained the informed consent from the parents or legal guardians, as well as the assent from the adolescent patient after thorough explanation of the study purpose and procedures.

Respondents were given numbered codes by the researcher and/or research assistant as they came. The socio-demographic data including patient's Age, Sex, Ethnicity, Current Education Status, Highest Parental Education and their health condition based on subspecialty were asked in the registration form then they proceeded with the interviewer-guided questionnaire. The tool used was the validated Transition Readiness Assessment Questionnaire (TRAQ) developed by David Wood et al. (6), which was adapted from East Tennessee State University Department of Pediatrics. This tool was chosen among the other readiness assessment tool since it has demonstrated adequate content and construct validity, as well as internal consistency and recommended as the best validated tool for its purpose (7). The TRAQ is a 20-item, 5-domain patient reported assessment of health and health care self-management skills which was recorded and analyzed on a 5-point scale basis. Each item is scored 1 to 5, with 1 being assigned for responses of “No, I do not know how” and a score of 5 assigned for responses of “yes, I always do this when I need to.” Among the 20 questions, two questions regarding health insurance application and coverage were omitted by the researcher since individuals who are 18 years old and below are still considered as dependents of their parents in health insurance policies in the Philippines. Hence, modifications were necessary to make the tool more appropriate in our actual setting, leaving us with a total of 18 questions included in this study. Since the TRAQ was modified, content validity was tested by three consultants- the first validator was an Epidemiologist, the second was a pediatrician, and the third was an Internist. The researcher used the Content Validation tool adapted from the graduate program of the school of natural sciences of a private university in Baguio City yielding an average score of 3.56 from the three validators, interpreted as **valid**. The modified questionnaire was also translated in Tagalog and Ilocano versions, with Reliability testing done using the Cronbach's coefficient of reliability with a result of 0.728 for the Tagalog version and 0.886 for the Ilocano version, both interpreted as **reliable**.

The TRAQ was designed to represent the five stages of change of the Transtheoretical Model which was organized into a 5-point ordinal response scale by Prochaska & DiClemente, 1986 (8). The scale of interpretation for the TRAQ response category and scores correspond to the five stages as seen in Table 1. Furthermore, since the study utilized a Likert Scale, the study also used the Information Literacy Education Implementation Readiness Scale with the scale of interpretation shown in Table 2 (9). Hence, an average score of 4 to 5 means that the subject is ready to transition and the health care planning from childhood to adulthood is adequate. However, a score of 3.99 and below meant that the participant was not ready to transition from child to adult health care, thus, implicating the need for a transition clinic or further assistance in health care planning.

**Table 1:** Stages of change model for TRAQ development by Prochaska, 1986.

Stage	Definition	TRAQ response category	TRAQ score
Precontemplation	Has no intention of taking action within the next 6 months	I do not need to do this	1
Contemplation	Intends to take action in the next 6 months	I do not know how but I want to learn	2
Preparation	Intends to take action within the next 30 days and has taken some behavioral steps in this direction	I am learning to do this	3
Action	Has changed behavior for less than 6 months	I have started doing this	4
Maintenance	Has changed behavior for more than 6 months	I always do this when I need to	5

**Table 2:** Information literacy education implementation readiness scale.

Mean rating	Interpretation	Readiness
5.00	Yes, I always do this when I need to	Ready
4.00–4.99	Yes, I have started doing this	
3.00–3.99	No, but I am learning to do this	Approaching readiness
2.00–2.99	No, but I want to learn	Developing readiness

The researcher and/or the research assistant collected the data while ensuring anonymity by utilizing numbered codes in the registration form at the same time double-checking that all questions were answered by the patients ensuring no drop-outs. The scores of the tests as well as the demographic data were recorded and encoded in a Microsoft Excel Data sheet on the same day that they were taken.

### Statistical analysis

The data gathered were encoded in a prepared spread sheet in Microsoft Excel and was statistically processed using SPSS version 20. Descriptive statistics using frequency and percentages were used to describe the demographic data of 15 to 18 years old adolescents. To determine the readiness of the adolescent patient towards transitioning from pediatric to adult clinics with regards to ability in managing medications, appointment keeping, tracking health issues, talking with providers and managing daily activities, the average mean scores as well as the standard deviation were computed. To determine if there is an association between the identified factors (such as the educational status of patients, education of their parents, and their kind of illness based on subspecialty) and the patient's readiness to transition to adult health care, One-way analysis of variance (ANOVA) using F-test was utilized to compare the MEANS across different categories of a variable. A *P*-value of ≤0.05 was considered statistically significant.

## Results

### Demography

A total of 63 respondents were analyzed and were grouped according to their Age, wherein one third of the respondents were 18 years old at 34.9%, and the remaining two-thirds are 15, 16 and 17 years old at 17.5%, 20.6% and 27% respectively. Majority of the respondents were females at 65.1%. For the ethnicity, 44.4% of these patients belongs to the Ethnic groups in Benguet which includes Kankana-ey, Ibaloi and Karao Tribe. On the other hand, 19% are Ilocano, 14.3% are Tagalog, and 7.9% are Pangasinense. The remaining 14.3% of the respondents belong to the Kalinga, Ifugao, and Kapampangan ethnic groups. For the current educational status of the patients, most of them were in High school at 73%, whereas 14.3% were already in college levels, 4.8% were still in Elementary, and 7.9% are not studying. The highest parental status of the respondents was also recorded showing that 41.3% of them were high school graduates, 28.6% finished College, and 19% finished Elementary level, while the remaining 11.1% had Post-graduate and Vocational Technical school courses. Almost half of these patients sought consult under General Pediatrics at 49.2% and the remaining half sought consults from the different subspecialty clinics as follows: Hematology-Oncology, Cardiology and Nephrology each had 9.5% of respondents, Allergology and Neurology both had 6.3%, Gastroenterology and Rheumatology had 3.2%, while Pulmonology and Endocrinology each had 1.6%.

### Factors affecting readiness

Among all Age groups, Sex and Ethnicity, these adolescents obtained an overall mean score of less than 4, denoting that they are not yet ready to transition to adult care settings. Furthermore, the respondents were not yet ready to transition regardless of their current educational status, parental educational status and their nature of illness, whether chronic or acute (Table 3).

**Table 3:** Standard deviation, mean and weighted mean scores per domain of transition readiness for all respondents according to factors affecting the study.

Factors affecting the study	Overall traq score mean (±SD)	Managing medications mean (±SD)	Appointment keeping mean (±SD)	Tracking health issues mean (±SD)	Talking with providers mean (±SD)	Managing daily activities mean (±SD)
Age						
15 years old	3.49 (0.51)	3.14 (0.98)	3.07 (0.73)	2.57 (0.86)	4.36 (0.60)	4.30 (0.75)
16 years old	3.72 (0.54)	3.29 (0.82)	3.57 (0.66)	2.87 (0.61)	4.27 (1.01)	4.59 (0.65)
17 years old	3.64 (0.49)	3.47 (0.92)	3.11 (0.89)	2.90 (0.52)	4.44 (0.66)	4.28 (0.64)
18 years old	3.77 (0.43)	3.49 (0.87)	3.49 (0.55)	3.02 (0.90)	4.52 (0.57)	4.30 (0.67)
**Weighted Mean**	**3** **.** **655**	**3** **.** **3475**	**3** **.** **31**	**2** **.** **84**	**4** **.** **3975**	**4** **.** **3675**
Sex						
Male	3.64 (0.54)	3.40 (0.88)	3.31 (0.69)	2.81 (0.86)	4.32 (0.78)	4.35 (0.74)
Female	3.69 (0.46)	3.37 (0.89)	3.34 (0.75)	2.91 (0.69)	4.48 (0.65)	4.36 (0.63)
**Weighted mean**	**3** **.** **665**	**3** **.** **385**	**3** **.** **325**	**2** **.** **86**	**4** **.** **4**	**4** **.** **355**
Ethnicity						
Benguet (Kankanaey, Ibaloi, Karao)	3.65 (0.48)	3.43 (0.82)	3.20 (0.70)	2.88 (0.79)	4.36 (0.78)	4.37 (0.66)
Ifugao	3.93 (0.37)	3.40 (1.00)	4.12 (0.52)	2.90 (0.38)	4.70 (0.45)	4.53 (0.19)
Kalinga	4.05 (0.55)	3.67 (0.94)	3.73 (0.70)	2.83 (1.13)	5.00 (0)	5.00 (0)
Tagalog	3.83 (0.44)	3.64 (0.95)	3.33 (0.87)	3.19 (0.78)	4.44 (0.53)	4.56 (0.37)
Pangasinense	3.65 (0.71)	3.30 (1.28)	3.12 (0.83)	2.75 (0.73)	4.80 (0.27)	4.27 (0.98)
Kapampangan	3.80 (−)	3.25 (−)	4.00 (−)	3.75 (−)	4.00 (−)	4.00 (−)
Ilocano	3.41 (0.38)	3.04 (0.86)	3.23 (0.54)	2.60 (0.65)	4.17 (0.81)	4.00 (0.80)
**Weighted mean**	**3** **.** **76**	**3** **.** **39**	**3** **.** **53**	**2** **.** **99**	**4** **.** **5**	**4** **.** **39**
Educational status						
Not studying	3.45 (0.21)	2.95 (0.33)	3.36 (0.17)	2.85 (0.78)	3.70 (0.67)	4.40 (0.83)
Elementary	3.21 (0.61)	2.58 (0.72)	2.93 (1.01)	2.42 (0.63)	4.33 (0.29)	3.78 (1.02)
High school	3.73 (0.49)	3.52 (0.93)	3.33 (0.74)	2.91 (0.74)	4.48 (0.72)	4.42 (0.61)
College	3.65 (0.44)	3.19 (0.72)	3.42 (0.80)	2.89 (0.87)	4.56 (0.46)	4.19 (0.75)
**Weighted mean**	**3** **.** **51**	**3** **.** **06**	**3** **.** **26**	**2** **.** **7675**	**4** **.** **2675**	**4** **.** **1975**
Highest parental education						
Did not study	**–**	**–**	**–**	**–**	**–**	**–**
Elementary graduate	3.61 (0.62)	3.27 (0.97)	3.75 (0.73)	2.75 (0.87)	4.17 (0.98)	4.14 (0.78)
High school graduate	3.73 (0.48)	3.43 (0.92)	3.32 (0.58)	2.99 (0.75)	4.42 (0.61	4.51 (0.56)
College graduate	3.63 (0.50)	3.42 (0.91)	3.13 (0.86)	2.96 (0.74)	4.47 (0.67)	4.17 (0.73)
Post graduate	3.73 (0.25)	2.88 (0.18)	3.40 (0.57)	2.38 (0.88)	5.00 (0.00)	5.00 (0.00)
Vocational technical school graduate	3.62 (0.38)	3.45 (0.67)	3.08 (0.73)	2.50 (0.35)	4.60 (0.42)	4.47 (0.51)
**Weighted mean**	**3** **.** **664**	**3** **.** **29**	**3** **.** **336**	**2** **.** **716**	**4** **.** **532**	**4** **.** **458**
Subspecialty						
General pediatrics	3.75 (0.45)	3.54 (0.89)	3.30 (0.76)	2.85 (0.79)	4.61 (0.60)	4.46 (0.61)
Hematology-oncology	3.35 (0.43)	2.92 (0.63)	3.00 (0.78)	2.46 (0.51)	4.25 (0.69)	4.11 (0.81)
Cardiology	3.76 (0.46	3.33 (0.86)	3.50 (0.56)	3.29 (0.45)	4.25 (0.42)	4.45 (0.40)
Pulmonology	3.56 (0.00)	3.50 (0.00)	2.80 (0.00)	3.50 (0.00)	4.00 (0.00)	4.00 (0.00)
Allergology	4.01 (0.23)	3.75 (0.84)	3.65 (0.25)	3.31 (0.63)	4.75 (0.50)	4.59 (0.42)
Neurology	3.10 (0.32)	2.31 (0.69)	3.00 (0.75)	2.63 (0.32)	3.63 (1.18)	3.92 (0.99)
Nephrology	3.57 (0.62)	3.38 (0.86)	3.57 (0.95)	2.63 (0.93)	4.17 (0.82)	4.11 (0.83)
Endocrinology	4.11 (0.00)	4.75 (0.00)	3.80 (0.00)	3.50 (0.00)	4.50 (0.00)	4.00 (0.00)
Gastroenterology	3.69 (0.71)	3.50 (0.35)	3.70 (0.71)	3.25 (0.35)	4.00 (1.41)	4.00 (1.41)
Rheumatology	3.76 (0.76)	3.00 (1.41)	3.30 (0.99)	2.75 (1.06)	4.75 (0.35)	5.00 (0.00)
**Weighted mean**	**3** **.** **666**	**3** **.** **398**	**3** **.** **362**	**3** **.** **017**	**4** **.** **291**	**4** **.** **264**
**Overall weighted mean**	**3** **.** **64**	**3** **.** **31**	**3** **.** **33**	**2** **.** **85**	**4** **.** **40**	**4** **.** **33**
Interpretation based on the information literacy education implementation readiness scale	Approaching readiness	Approaching readiness	Approaching readiness	Developing readiness	Ready	Ready

Bold values denote the over all weighted mean per factor per domain.

These adolescents were deemed Ready to transition in terms of Talking with providers and managing daily activities with weighted mean scores of 4.40 and 4.33 respectively. However, in terms of Managing medications and Appointment Keeping, the adolescents' scores had weighted mean scores of 3.31 and 3.33 respectively, which is interpreted as still Approaching readiness. Whereas their weighted mean score for Tracking health issues was 2.85 only, interpreted as still in the stage of Developing readiness. Overall, the average scores for all domains from all the respondents yielded 3.64, which means that the adolescents seen in our Pediatric clinics are still in the stage of Approaching readiness.

### Transition readiness per TRAQ domain

With regards to managing medications, one fourth of respondents do not know how to fill or ask for prescriptions if they need to. One third of respondents do not know what to do in case of medication reactions but are willing to learn how. Majority of respondents know how to take medications correctly on their own. Two-thirds of the patients are already reordering medications before they run out (Table 4).

**Table 4:** Percentage distribution of the respondents according to their responses per domain of transition readiness from pediatric to adult clinics.

	No, I do not know how # (%)	No, but I want to learn # (%)	No, but I am learning to do this # (%)	Yes, I have started doing this # (%)	Yes, I always do this when I need to # (%)
Managing medications					
1. Do you fill a prescription if you need to?	16 (25.4)	13 (20.6)	12 (19.0)	11 (17.5)	11 (17.5)
2. Do you know what to do if you are having a bad reaction to your medications?	6 (9.5)	20 (31.7)	12 (19.0)	11 (17.5)	14 (22.2)
3. Do you take medications correctly and on your own?	1 (1.6)	6 (9.5)	3 (4.8)	27 (42.9)	26 (41.3)
4. Do you reorder medications before they run out?	10 (15.9)	9 (14.3)	7 (11.1)	15 (23.8)	22 (34.9)
Appointment keeping					
5. Do you call the doctor's office to make an appointment?	5 (7.9)	15 (23.8)	17 (27.0)	16 (25.4)	10 (15.9)
6. Do you follow-up on any referral for tests, check-ups or labs?	2 (3.2)	8 (12.7)	10 (15.9)	29 (46.0)	14 (22.2)
7. Do you arrange for your ride to medical appointments?	5 (7.9)	13 (20.6)	14 (22.2)	18 (28.6)	13 (20.6)
8. Do you call the doctor about unusual changes in your health (for example: allergic reactions)?	5 (7.9)	16 (25.4)	17 (27.0)	13 (20.6)	12 (19.0)
9. Do you manage your money & budget household expenses (For example: use checking/debit card)?	8 (12.7)	12 (19.0)	9 (14.3)	24 (38.1)	10 (15.9)
Tracking health issues					
10. Do you fill out the medical history form, including a list of your allergies?	7 (11.1)	15 (23.8)	14 (22.2)	15 (23.8)	12 (19.0)
11. Do you keep a calendar or list of medical and other appointments?	4 (6.3)	14 (22.2)	19 (30.2)	19 (30.2)	7 (11.1)
12. Do you make a list of questions before the doctor's visit?	13 (20.6)	24 (38.1)	15 (23.8)	10 (15.9)	1 (1.6)
13. Do you get financial help with school or work?	18 (28.6)	13 (20.6)	6 (9.5)	17 (27.0)	9 (14.3)
Talking with providers					
14. Do you tell the doctor or nurse what you are feeling?	2 (3.2)	4 (6.3)	2 (3.2)	24 (38.1)	31 (49.2)
15. Do you answer questions that are asked by the doctor, nurse or clinic staff?	0 (0)	1 (1.6)	1 (1.6)	20 (31.7)	41 (65.1)
Managing daily activities					
16. Do you help plan or prepare meals/food?	1 (1.6)	3 (4.8)	4 (6.3)	22 (34.9)	33 (52.4)
17. Do you keep home/room clean or clean-up after meals?	1 (1.6)	2 (3.2)	5 (7.9)	18 (28.6)	37 (58.7)
18. Do you use neighborhood stores and services (for example: grocery stores and pharmacy stores)?	0 (0)	3 (4.8)	5 (7.9)	22 (34.9)	33 (52.4)

In terms of appointment keeping, less than half are able to make an appointment and do follow-up for consultations and laboratory results on their own. Likewise, independently arranging for transportation for consultation appointments is only being practiced by less than half of the respondents. When it comes to occurrence of any allergic reactions or unusual changes in their health, only one third will call their doctor for help, while the remaining will not. Managing money and budgeting for household expenses are already being done by half of the adolescents independently.

Responses with regards to tracking health issues showed varied responses for each question. Properly filling up the medical history forms and making a list of questions before doctor visits had majority of “No, but I want to learn” responses, while keeping a calendar of appointments is not yet done by two-thirds of the patients. Majority of adolescents do not get financial help from school or work.

Talking with health care providers is also a task that these adolescents need to practice. Telling or verbalizing to the nurse or doctor what he or she is feeling is already being practiced by majority as well as answering to the health care staffs when prompted with questions about their health.

Lastly, managing daily activities include the ability to independently plan/prepare meals, keep own room's clean, and go to nearby stores or obtain basic health treatments such as from the pharmacies. For this domain, more than half of the respondents are already doing the aforementioned tasks.

### Association of factors and readiness to transition

One-way analysis of variance (ANOVA) using *F*-test was utilized to compare the MEANS across different categories of a variable wherein a *P*-value of <0.05 was considered statistically significant. However, in this study the generated *P*-values in the statistical tests assessing the significant association of some factors in relation to the respondent's readiness to transition from pediatric to adult care were more than 0.05 for all variables.

## Discussion

Of the many changes happening in the adolescents, one of the most important is the shift of cognitive functioning from concrete to abstract type of thinking. Improvement in their cognitive development results from an interplay of brain maturation coupled with the influence of the environment. Adolescents in the middle stage of development experience various emotional and social changes which include increased self-involvement, and drive for independence. During this period, they are now more able to appreciate the impact of their actions on their health, and in turn, have a clearer understanding of their illness and management of their own health care. Having such abilities were considered in choosing the age group to be assessed in this research study.

In terms of progression of health care, changing from a setting of pediatric, parent-supervised health care to a more independent patient-centered adult health care system is a big step for adolescent patients (3). However, some Pediatricians were reported to sometimes ignore the adolescent's growing independence in contrast to adult clinicians who encourage immediate responsibility for their own health care (10). This leads to the perception of feeling lost in adult health care settings, which further decreases follow up rates and compliance to medications (11). Gaps in evaluation and treatment such as these prompted the researcher to assess the status of adolescents seen in our institution with regards to readiness to transition.

The Transition Readiness Assessment Questionnaire in this study was only used as a baseline evaluation, with no intervention to improve self-management skills and re-evaluation done because our institution does not yet have a structured transition program. This study is similar to sixteen studies which measured the outcomes of patients who were evaluated under clinics which do not have existing transition policies (12), hence it is recommended that TRAQ score be evaluated over time so it could be more predictive of transition ability.

The generated *P*-values in the statistical tests assessing the significant association of some factors in relation to the respondent's readiness to transition from pediatric to adult care were more than 0.05 for all variables. This implies that the readiness of the adolescents towards transitioning from pediatric to adult clinics is not significantly associated to the Sex, Age, Ethnicity, Educational status, Highest parental education and Kind of illness based on Subspecialty. Furthermore, this implies that these adolescents have the same level of readiness across the different variables. The results of this study are similar to the study done by Jensen et al., wherein the baseline TRAQ scores were not significantly related to age groups, sex, race, or disease specialty (all *P*-values >0.05.) Hence, it is still suggested that significant efforts in research studies, policy changes, advocacy, and education of health care providers and families are important to ensure the best transition preparation and adult outcomes for youth with special needs from all ethnic backgrounds (13).

In this study, two-thirds of them were 15, 16 and 17 years old while the remaining one-third were 18 years old. In our country, the adolescents and youth tend to seek health care less than those who are either younger or older, probably because they are no longer affected by common childhood illnesses and have yet to encounter health problems that are common in the older population (14). These findings coincide with the result of this since all age group had TRAQ Mean scores recorded less than 4, suggesting that they are not yet ready to transition to the adult clinics. This is also similar to a study done by Chan J.T. et al. wherein their overall Scores across all age group are less than 4 except for those who are 18–19 years old (15).

Majority of the respondents were females. In a survey done by Yu et al., sex difference could affect successful transition wherein their data suggest that young women are more likely to make a successful transition to adult health care compared to young men (16). However, in this study, both sexes had an overall TRAQ Mean scores of 3.64 for males and 3.69 for females, implying that both were still in the stage of Approaching Readiness.

Ethnicity is important to consider in terms of analyzing the health seeking behaviors. Some groups are still considered disadvantaged both in access to health care and education owing to their geographical location. In this study, almost half of these patients belong to the Ethnic groups in Benguet which includes Kankana-ey, Ibaloi and Karao Tribe. Other respondents are Ilocano, Tagalog, Pangasinense, Kalinga, Ifugao, and Kapampangan ethnic groups. In a study by George et al., cultural expectations and social opportunity structures have an influence with regards to the timing and patterning of role entries and exits during the transition to adulthood (17). Since this is the first study done in the region, it showed that the overall TRAQ mean scores for all ethnic group classifications ranges from 3.41 to 4.05, denoting that all respondents were still in the stage of Approaching readiness except the Kalinga ethnic group. Hence, it is still suggested that significant efforts in research studies, policy changes, advocacy, and education of health care providers and families are important to ensure the best transition preparation and adult outcomes for the youth from all ethnic backgrounds.

For the current educational status of the patients, most of them were in High school whereas the remaining are in College, Elementary, and some are not studying. However, regardless of the current educational status of patients, the overall TRAQ mean score ranged from 3.61 to 3.73, which means that they were not yet ready to transition to adult health care.

On the other hand, the highest parental status of the respondents were High school graduates, while others finished College, Elementary level, Post-graduate and Vocational Technical school courses. In a study by Sheng et al., there is strong evidence that Parental education can help in an easier transition of patients to adult health care (18). However, in this study, there was no notable difference in transition readiness regardless of parental education since the overall scores were below 4.0. Regardless of the parental educational status, the primary care givers should still be involved in health care planning to maximize the chance of transitioning the patients successfully. This is to avoid the possibility of failed transition such as the results in a study done by Lotsein et al., wherein a national survey of parents or guardians of youth with special health care needs (YSCHN) have found out that only 50% report having discussed transition with their adolescent's physician (13) and only 30% had a plan for addressing those needs (19).

Almost half of these patients sought consult under General Pediatrics and the remaining half sought consults from the different subspecialty clinics. The process of preparing youth for the move to adult care has been studied in diverse patient populations, including cystic fibrosis (20), congenital heart disease (21), diabetes (22), asthma (23), cerebral palsy (24), and mental health conditions (25, 26). Hence, in other similar studies, transition clinics have been done for patients mostly under subspecialty care who require chronic health care management. Although literature support the importance of transition clinics in the subspecialty areas, it is also essential to prepare for transition those patients under general pediatrics as well (White et al. 2018). In this study, almost half of these patients sought consult under General Pediatrics and the remaining half sought consults from the different subspecialty clinics. Results showed that among the patients seen both in the subspecialty and general pediatrics clinic, there was no difference between subspecialty in terms of readiness to transition except for patients seen in Allergology and Endocrinology Clinics who had an overall TRAQ mean score of 4.01 and 4.11 respectively.

For the specific percentage distribution of the respondents according to their readiness towards transitioning from pediatric to adult clinics, with regards to Managing Medications, one fourth of respondents do not know how to fill or ask for prescriptions if they need to, while one third already knows how to. One third of respondents do not know what to do in case of medication reactions but are willing to learn how, while almost one fourth already knows what to do. Majority of respondents know how to take medications correctly on their own at 84.2% with only 1.6% who still do not know. More than half are already reordering medications before they run out while the remaining are not yet practicing it but shows willingness to learn. The results were comparable with the report done by Valenzuela and Van Staa wherein Pediatricians were reported to sometimes ignore the adolescent's growing independence in contrast to adult clinicians who encourage immediate responsibility for their own health care. This often leads to the perception of feeling lost in adult health care settings, which further decreases follow up rates and compliance to medications (11).

In terms of Appointment keeping, one fourth are still learning to call their doctors to schedule an appointment, while 15% are already making an appointment on their own. Almost half of respondents have started doing follow-up for consultations and laboratory results, while 3.2% still do not. Independently arranging for transportation for consultation appointments are already being practiced by almost half of the respondents however less than 10% are not yet practicing it. When it comes to occurrence of any allergic reactions or unusual changes in their health, majority still do not call their physicians for assistance. Managing money and budgeting for household expenses are already being done by more than half of the adolescents independently.

Based on the 2008 National Demographic and Health Survey (NDHS) of our country, the most common reasons for visits to local health facilities were illness and injury (67.6%) and medical check-ups (28.1%). In this study, patients are recorded to have difficulties in Appointment keeping probably because it is similar to the reports that among women aged 15–19, 78.5% cited having at least one problem accessing health care, with lack of money for treatment being the most common reason (56.8%) (14).

Responses with regards to tracking health issues showed varied responses for each question. In properly filling up the medical history forms and making a list of questions before doctor visits majority are “Not yet ready”, while keeping a calendar of appointments both had one third for “No, but I am learning” and “Yes, I have started doing”. Majority of adolescents do not prepare a list of questions prior to consultations and/almost the same number do not get financial help from school or work. This domain showed the lowest score in terms of readiness to transition. The results are also in line with the National Survey of Children's Health done in the United States, wherein they found out that only 15% of youth receive assistance from their health care professionals in planning the transition from pediatric to adult care (13).

Talking with health care providers is also a task that these adolescents need to practice. Telling or verbalizing to the nurse or doctor what he or she is feeling is already being practiced by half of these adolescents however less than 5% are still unlikely to voice out their feelings. Furthermore, there is a high percentage of the population that answers to the health care staffs when prompted with questions about their health. Overall, majority are ready in terms of talking with health care providers.

Lastly, the 5th domain of TRAQ includes Managing Daily Activities which had a consistently more than half of respondents being able to independently plan or prepare meals, keep own room's clean, and go to nearby stores or obtain basic health treatments such as from the pharmacies. Overall, these patients are deemed ready in terms of Managing Daily Activities.

With these results, it is highly recommended that transition planning should be a standard part of providing care for all youth and young adults, and every patient should have a transition plan regardless of his or her specific health care needs as also stated in the study of Cooley et al. (27). The important benefits cited in establishing adequate transition to health care of these adolescents include reduction in medical complications, improvement in patient-reported outcomes, greater adherence to care, positive patient experience and lower cost of health care (28). This study will serve as an initial assessment of the preparedness of adolescents to manage the various aspects of their own health care, so we can help them get ready in terms of independent and effective health care management. Having a baseline evaluation of the patients' readiness to transition is of great importance in order to understand other gaps in knowledge and skills to transition and to help in the formulation of guidelines and policies for an easier transition to adult health care system. Since the institution does not yet have a transition clinic or program, the knowledge gained shall help both Pediatricians and Adult Health Care providers, to establish a platform for assisting the adolescents in their assumption of bigger roles and responsibilities for their individual health care. The process will start with the development of a transition policy with re-evaluation of readiness to transition, and later on, its dissemination to all families seeking consult in our institution, so they can better understand that transition planning will be part of the patient's overall health care management.

## Conclusion

In summary, this study showed that adolescent patients, 15 to 18 years of age, who had both acute and chronic illnesses, were not yet ready to transition from pediatric to adult health care. In addition, there were no factors identified that affects the readiness to transition to adult health care. Looking at the five domains of readiness, these adolescents were ready in terms of Talking with health care providers and Managing Daily activities; however, they still need assistance in terms of Managing medications, Appointment keeping and Tracking health issues. The results were highly suggestive of the benefits of having a transition clinic to both patients and their families in terms of planning of health care, both long and short term. Provision of a platform for assisting the adolescents in their assumption of bigger roles/responsibilities for their own health care is necessary to ensure proper transitioning to adult health care. Development of a transition policy and, later on, its dissemination to all families are important so they can understand better that transition planning will be part of both acute and chronic care management. Since the institution does not have transition clinics or programs, this study will be helpful for the Pediatricians, Internists and the institution itself in promoting and improving the standard of care for these adolescents who are in need for such platform.

## Strengths and limitations

### Strengths

Realizing that the adolescent patients seen in our institution are not ready to transition to adult health care, implementation of a transition care clinic or program would be of great benefit to the patients, their parents, and the health care givers as well. This baseline evaluation of the patients' readiness to transition is of great importance in order to understand other gaps in knowledge and skills to transition and to help in the formulation of guidelines and policies for an easier transition to adult health care system.

### Limitations

Since this study had been done during the COVID 19 pandemic wherein strict health care restrictions had been in effect. Further study in another institution and larger population is recommended since this study was conducted in one institution and only in the Department of Pediatrics Outpatient Department for four months.

The institution can benefit early on by starting to implement a Transition Program since establishing a formal physical Transition Clinic will take time. It is also recommended to continue assessing the readiness to transition of all adolescent patients seen in our institution, and to do a re-evaluation of the effectiveness of transition clinic after implementation of transition clinics since it has been documented that there was a significant impact on patients' outcomes post-transition preparing the adolescent and young adult patients. Since both our general and subspecialty clinics cater to all patients, the frequency of visits to the Outpatient department should also be included in the factors affecting transition readiness since it may also affect the adolescent's heath care practices. Those requiring urgent admission or referral to other departments were not included in the study, however, they should also be included once they are stable enough to answer the questionnaire. The perception of the parents or guardians can also be included since transition does not involve the patients alone, but most importantly, their caregivers who stand as the main decision makers in terms of overall care.

Lastly, it is also recommended to continue assessing the readiness to transition of all adolescent patients seen in our institution, and to do a re-to evaluation of the effectiveness of transition clinic after implementation of transition clinics since it has been documented that there was a significant impact on patients' outcomes post-transition preparing the adolescent and young adult patients. Coordinating the transition clinics with the various adult health care services will be a very important step to ensure the success of the transition clinic.

## Data Availability

The original contributions presented in the study are included in the article, further inquiries can be directed to the corresponding authors.
