# Supplementary figures and images for: Transition readiness of adolescents to adult health care
Source: Front Pediatr. 2023 Jul 31;11:1204019. doi: 10.3389/fped.2023.1204019 (PMC10426738; doi:10.3389/fped.2023.1204019)

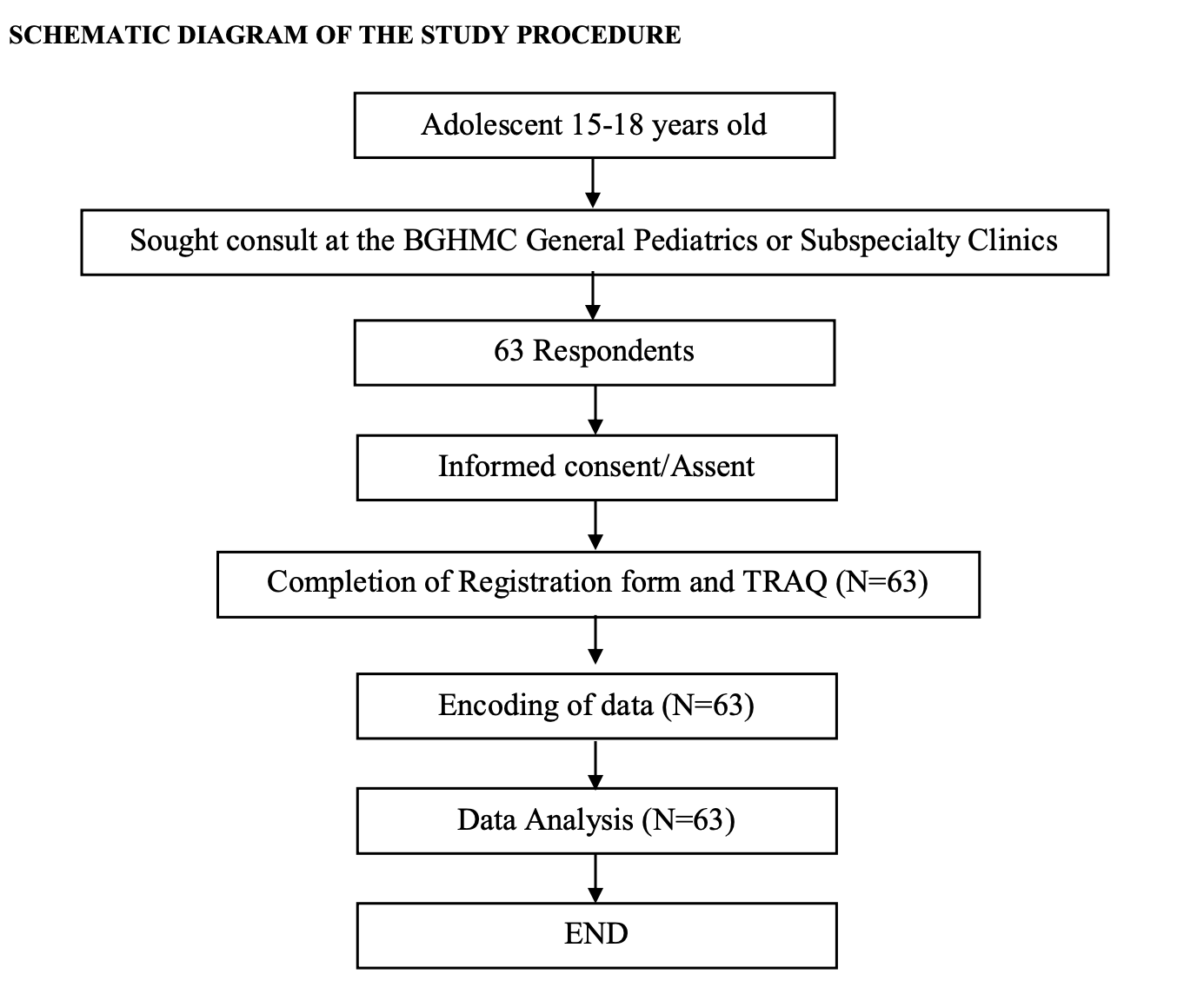

Supplement: Supplementary file 1 [file Image1.png]
